# Evidence and Role for Bacterial Mucin Degradation in Cystic Fibrosis Airway Disease

**DOI:** 10.1371/journal.ppat.1005846

**Published:** 2016-08-22

**Authors:** Jeffrey M. Flynn, David Niccum, Jordan M. Dunitz, Ryan C. Hunter

**Affiliations:** 1 Department of Microbiology & Immunology, University of Minnesota, Minneapolis, Minnesota, United States of America; 2 Division of Pulmonary, Allergy, Critical Care & Sleep Medicine, University of Minnesota, Minneapolis, Minnesota, United States of America; Ohio State University, UNITED STATES

## Abstract

Chronic lung infections in cystic fibrosis (CF) patients are composed of complex microbial communities that incite persistent inflammation and airway damage. Despite the high density of bacteria that colonize the lower airways, nutrient sources that sustain bacterial growth *in vivo*, and how those nutrients are derived, are not well characterized. In this study, we examined the possibility that mucins serve as an important carbon reservoir for the CF lung microbiota. While *Pseudomonas aeruginosa* was unable to efficiently utilize mucins in isolation, we found that anaerobic, mucin-fermenting bacteria could stimulate the robust growth of CF pathogens when provided intact mucins as a sole carbon source. 16S rRNA sequencing and enrichment culturing of sputum also identified that mucin-degrading anaerobes are ubiquitous in the airways of CF patients. The collective fermentative metabolism of these mucin-degrading communities *in vitro* generated amino acids and short chain fatty acids (propionate and acetate) during growth on mucin, and the same metabolites were also found in abundance within expectorated sputum. The significance of these findings was supported by *in vivo P*. *aeruginosa* gene expression, which revealed a heightened expression of genes required for the catabolism of propionate. Given that propionate is exclusively derived from bacterial fermentation, these data provide evidence for an important role of mucin fermenting bacteria in the carbon flux of the lower airways. More specifically, microorganisms typically defined as commensals may contribute to airway disease by degrading mucins, in turn providing nutrients for pathogens otherwise unable to efficiently obtain carbon in the lung.

## Author Summary

Persistent CF lung infections are composed of hundreds of microbial taxa whose interactions contribute to respiratory failure. Despite their importance, the complex interplay between the lung microbiota and host environment is poorly understood. For example, the nutrients that sustain bacterial growth *in vivo*, and how those nutrients are derived, are not well characterized. We reveal that a subset of CF microbiota is capable of fermenting mucins for carbon and energy which, in-turn, can support the carbon demands of other respiratory pathogens in co-culture. Moreover, we show that metabolites consistent with mucin fermentation are abundant within airway secretions, highlighting a potential key role for fermentative metabolisms in CF lung disease. A thorough understanding of pathogen ecology in the CF airway and the nutritional dynamics that sustain their growth will have important implications for the design of new therapeutic strategies and the management of disease progression.

## Introduction

Mutations in the gene encoding the cystic fibrosis transmembrane conductance regulator (CFTR) protein cause an imbalance of ion transport that leads to mucus hyperviscosity and impaired mucociliary clearance [1]. Within the airways, prolonged residence time of mucus provides a stagnant nidus for chronic bacterial infections–the predominant cause of mortality in CF patients [2]. Traditionally, culture-based studies have focused on a small number of taxa associated with CF lung disease (*e*.*g*. *Pseudomonas aeruginosa*, *Staphylococcus aureus*), however, culture-independent surveys of the CF lung microbiome have revealed a far more complex bacterial community than previously appreciated [3–5]. While the temporal dynamics of these communities and their association with disease states have been studied in detail, the *in vivo* host environment, and microbial metabolism therein, is relatively understudied.

For example, the means by which CF pathogens obtain sufficient energy for growth is not known. Bacterial numbers within the CF lung can reach 10^8^−10^9^ cells gm^-1^ of sputum [6], which are comparable to densities found within the distal colon [7]. However, unlike the gut where dietary sources provide a constant influx of nutrients, carbon within the airways must be predominately host-derived. The respiratory tract contains a number of host compounds that can be used by microbes as nutrient sources, including immunoglobulins, cytokines, defensins and lactoferrin [8,9], yet these are unlikely to be present at concentrations to support the dense microbiota of the CF airways [10–13]. Additionally, studies of CF sputum from adult patients have shown an abundance of small molecules that can support the growth of pathogens *in vitro*, including sugars, fatty acids, phospholipids, and amino acids [14–17]. However, the mechanism by which these compounds reach high abundance in airway mucus remains poorly defined.

The accumulation of mucus secretions in the CF airways represents an abundant nutrient source. The major macromolecular constituents of mucus, mucins, are a large reservoir of both carbon and nitrogen, and have been measured at concentrations of up to 10 g L^-1^ in sputum [18]. Mucins are high molecular weight (2–20 x 10^5^ Da) glycoproteins composed of an amino acid backbone with O-linked oligosaccharide side chains that form 50–90% of the molecular mass [19]. Carboxyl and sulfate groups decorate their terminal sugars conferring a net negative charge, while terminal cysteine-rich domains form disulfide bonds with neighboring polymers, forming a highly cross-linked gel-like structure that is resistant to rapid bacterial degradation [20].

Despite their recalcitrance, mucins are a main nutrient source for niche-specific microbiota of the gut and oral cavity. For example, oral streptococci produce a variety of glycolytic and proteolytic enzymes that liberate bioavailable carbohydrates from salivary glycoproteins [21]. Few single species are known that can completely degrade mucins when grown in monoculture [22], though specific consortia of oral bacteria have been shown to co-operatively degrade both the polysaccharide and peptide structures of salivary mucins [21,23]. In turn, these primary degraders are thought to modify the nutritional landscape of the oral cavity and stimulate the growth of secondary colonizers [24]. Similar interactions between commensal gut microbiota and the mucosal layer of the large intestine are well known [25]. By contrast, very little is known about the degradation of airway mucins by opportunistic pathogens and their role as a nutrient source *in vivo* [26].

Here, we investigated the role of mucins in the carbon flux of the CF airways and characterized their potential to stimulate pathogen growth. Our results demonstrate that *P*. *aeruginosa* uses mucins inefficiently in monoculture. This lack of growth raised the question: can other members of the CF microbiota degrade mucins and alter the nutritional reservoir available for pathogens? We subsequently found that co-culture of pathogens with an anaerobic bacterial consortium composed of taxa commonly found in the lower airways [3,5,27,28] can facilitate robust pathogen growth using mucins as a sole carbon source. We also confirmed that fermentation-derived metabolites are abundant within expectorated CF patient sputum, consistent with previous studies [14,29]. Finally, we found that genes required for the catabolism of mucin fermentation byproducts are highly expressed by *P*. *aeruginosa in vivo*. Taken together, these data support a central ecological role for commensal anaerobes in the nutritional dynamics of the lower airways and the progression of CF lung disease.

## Results

### *Pseudomonas aeruginosa* utilizes mucin inefficiently as a sole carbon source

**Fig 1:**
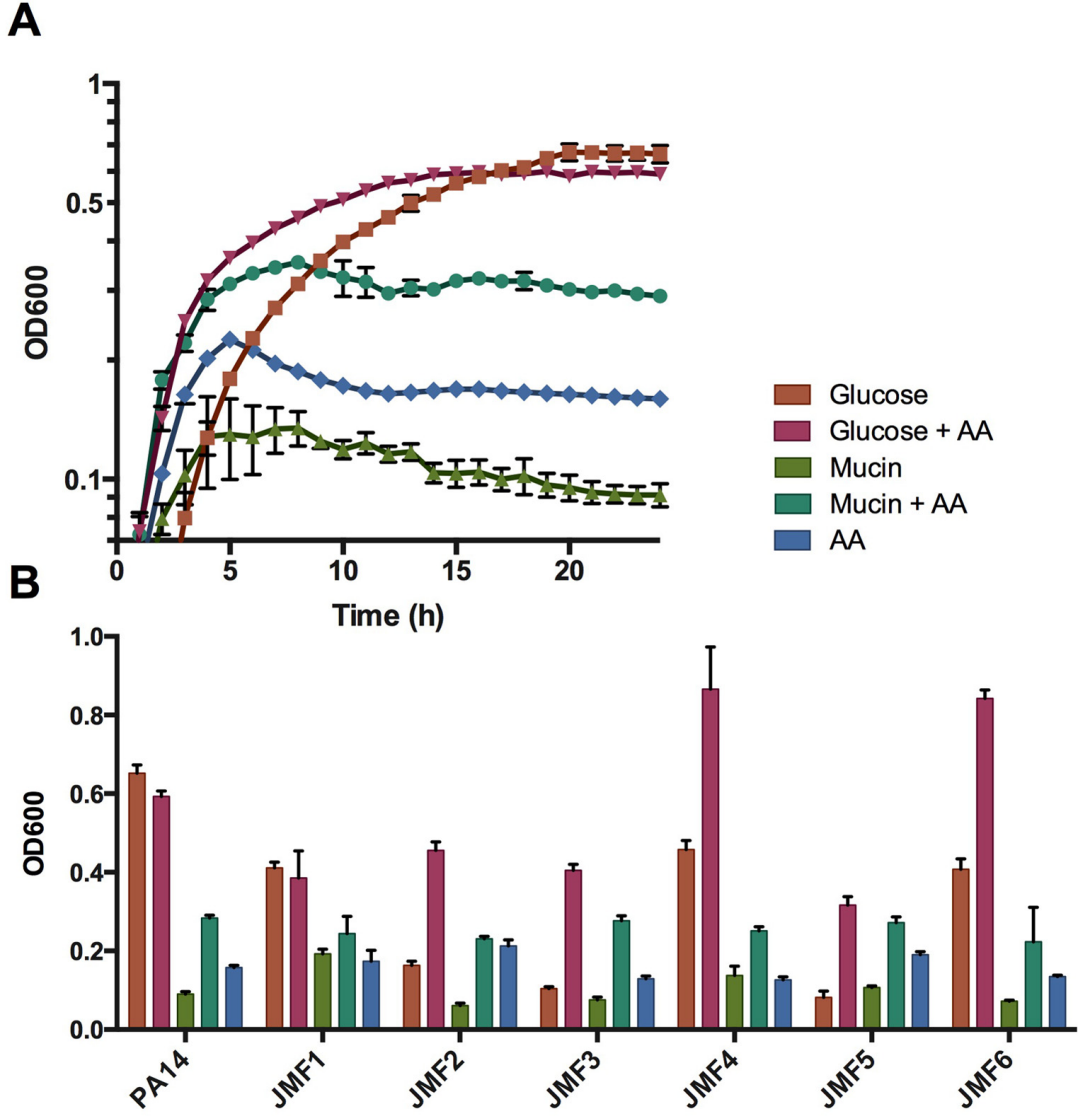
*P*. *aeruginosa* growth on mucin. (A) *P*. *aeruginosa* PA14 grown with mucin and glucose (+/- amino acid supplement). Data are presented as mean values from four independent measurements. (B) *P*. *aeruginosa* PA14 and clinical isolates grown with mucin and glucose (+/- complete amino acids supplement) for 48h. Data are shown as mean +/- SEM of endpoint culture optical density (600nm).

We first wanted to determine if mucins alone could sustain the growth of *P*. *aeruginosa*. To do so, we assayed strain PA14 for growth in a defined minimal medium containing intact mucins from porcine gastric mucin (PGM) as the sole carbon source (Fig 1A). PGM was first dialyzed and filtered to remove impurities and small metabolites that could potentially support growth (see Methods). Interestingly, 15g L^-1^ of purified mucins (sugar equivalent of ~65mM glucose assuming 80% sugar by weight) only resulted in a moderate OD_600_ gain of 0.1 after 24 h. By contrast, PA14 grew to 0.65 OD on glucose (13mM) alone, which underscores the inability of PA14 to efficiently break down and utilize PGM. Recognizing that PGM and human respiratory mucins (MUC5AC and MUC5B) are structurally diverse [30], we also assayed *Pseudomonas* growth in a minimal medium containing purified MUC5B as the sole carbon source (S1 Fig). PA14 reached a two-fold lower density when utilizing MUC5B relative to PGM, suggesting that *P*. *aeruginosa* PA14 cannot efficiently utilize complex mucin glycoproteins, including the most abundant mucin of the lower airway.

Given that PA14 was isolated from a burn wound, we then reasoned that *P*. *aeruginosa* clinical isolates derived from a mucin-rich sputum environment would have enhanced ability to degrade and utilize mucins as a growth substrate. To test this, clinical isolates derived from CF patients at various stages of disease were grown in minimal mucin medium. Growth yield was assayed long past the onset of stationary phase (48h) to account for slow growth phenotypes. Under these conditions, each isolate achieved a moderate density on PGM, comparable to PA14 (final OD_600_ between 0.06 and 0.19)(Fig 1B). Growth on mucin was also compared to growth with glucose alone, in addition to growth with a complete amino acid supplement to account for auxotrophy, a common trait among CF lung isolates [31]. Indeed, isolates JMF2, JMF3, and JMF5 grew poorly on glucose, suggesting auxotrophies that were corrected by the supplement. The ability of these isolates to grow on glucose supplemented with amino acids to a greater extent than the more nutrient-dense mucin medium suggests that the lack of robust growth on PGM was not due to slow-growth phenotypes. Rather, the fact that each isolate grew when sufficient carbon was made available to them demonstrates that *P*. *aeruginosa* has a general inability to efficiently utilize complex mucin glycoproteins in monoculture.

### Saliva-derived mucin-fermenting bacteria support the growth of opportunistic pathogens *in vitro*

The inefficiency of *P*. *aeruginosa* to use mucins as a growth substrate motivated us to consider recent culture-dependent and culture-independent studies of CF microbiota for insights into the carbon flux of the airways. Notably, obligately anaerobic taxa have gained recent attention for their abundance in expectorated sputum, bronchoalveolar lavage fluid and explanted lungs [5,27,28,32–34]. A role for these organisms in CF disease has not yet been established; however, some have been characterized for their ability to degrade and ferment salivary mucins in the oral cavity [21,23,35]. Based on these observations, we hypothesized that oral anaerobes, once aspirated into the lower airways, could alter the nutrient pool by degrading respiratory mucins. More specifically, we predicted that the degradation and fermentation of mucin glycoproteins would liberate sugars, amino acids and short chain fatty acids (SCFAs), all of which are abundant components of CF sputum that are readily utilizable by *P*. *aeruginosa* [36].

**Fig 2:**
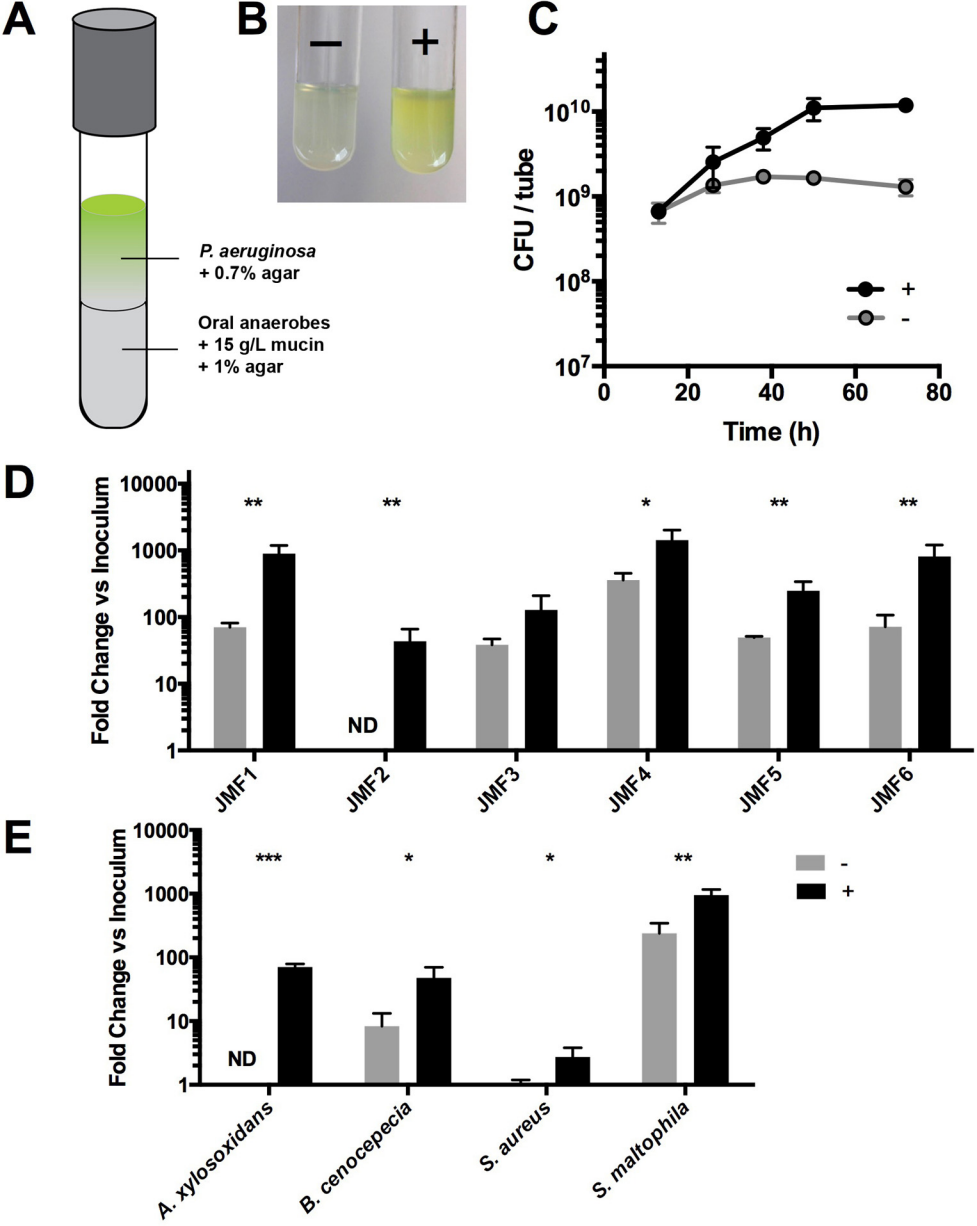
Oral-derived mucin fermenters support pathogen growth using mucin as the sole carbon source. (A) A saliva-derived bacterial consortium was grown anaerobically in agar with mucins and overlaid with an immobilized *P*. *aeruginosa* culture (inoculum = 4×10^5^ CFU) in an agar gel without mucins. (B) After 24h, only co-cultures containing mucin fermenting anaerobes (+) enabled robust *P*. *aeruginosa* growth and the producton of blue-green pigment characteristic of PA14. (C) Over a 72h time course, co-cultures containing mucin fermenting anaerobes enabled an order of magnitude higher growth of *P*. *aeruginosa* relative to tubes lacking the fermenting innoculum (p = 0.007). (D) PA14 and other clinical isolates of *P*. *aeruginosa*, and (E) various other CF pathogens grow to a higher density in the presence of mucin fermenters when mucin is the sole carbon source. Data are presented as mean values +/- SEM from three biological replicates. (ND = not detectable). Inoculum densities are shown in S2 Table.

To test whether fermentative bacteria are able to generate metabolites from mucin that could simultaneously stimulate CF pathogen growth, a saliva-derived bacterial community was first enriched on PGM (S2 Fig). This enrichment culture was then used to inoculate an anaerobic minimal mucin medium supplemented with 1.0% agar to mimic a high-viscosity sputum gel [37] (Fig 2A). Once the lower agar phase containing the mucin-enriched bacterial community had solidified, PA14 was suspended in buffered 0.7% agar medium without mucin (*i*.*e*. no carbon source) and was placed in the upper portion of the tube. This experimental setup a) establishes an oxygen gradient allowing anaerobes to grow, and b) restricts the movement of microbes but allows metabolites to freely diffuse. Under these conditions, *P*. *aeruginosa* would be expected to achieve a higher cell density if provided with diffusible growth substrates from the lower phase.

Co-culture growth was monitored over a 72h period. After 24h, turbidity was noticeable in the lower phase and a diffusible blue-green pigment (pyocyanin) characteristic of *P*. *aeruginosa* growth was observed throughout the co-culture tube (Fig 2B). By contrast, no observable pigment was produced in the absence of oral anaerobes. Colony counts of the upper phase revealed that *P*. *aeruginosa* reached its maximum density after 48h of co-culture (Fig 2C), and achieved an order of magnitude (10X) increase (p = 0.007) in cell density relative to the tubes in which oral anaerobes were omitted. These data demonstrate that while PA14 can achieve a moderate density using PGM as a sole growth substrate (consistent with Fig 1A), its growth and production of a known virulence factor is significantly enhanced via mucin breakdown and cross-feeding by oral-associated anaerobes.

We then tested the growth of several *P*. *aeruginosa* clinical isolates in addition to the CF-associated pathogens *Achromobacter xylosoxidans*, *Burkholderia cenocepecia*, *Stenotrophomonas maltophilia* and *Staphylococcus aureus* using the agar co-culture model (Fig 2D and 2E). For each bacterium, a notable increase in growth over the inoculum was observed in the presence of mucin fermenters after 48h. With the exception of JMF3, the final cell density was significantly higher for each co-culture relative to monoculture tubes in which anaerobes were omitted. Collectively, these data support the hypothesis that oral-derived microbiota can serve as primary mucin degrading organisms, in turn liberating metabolites that stimulate the growth of *P*. *aeruginosa* and other CF lung pathogens.

### Oral-associated bacteria are present in sputum and are enriched in anaerobic mucin fermentation cultures

**Fig 3:**
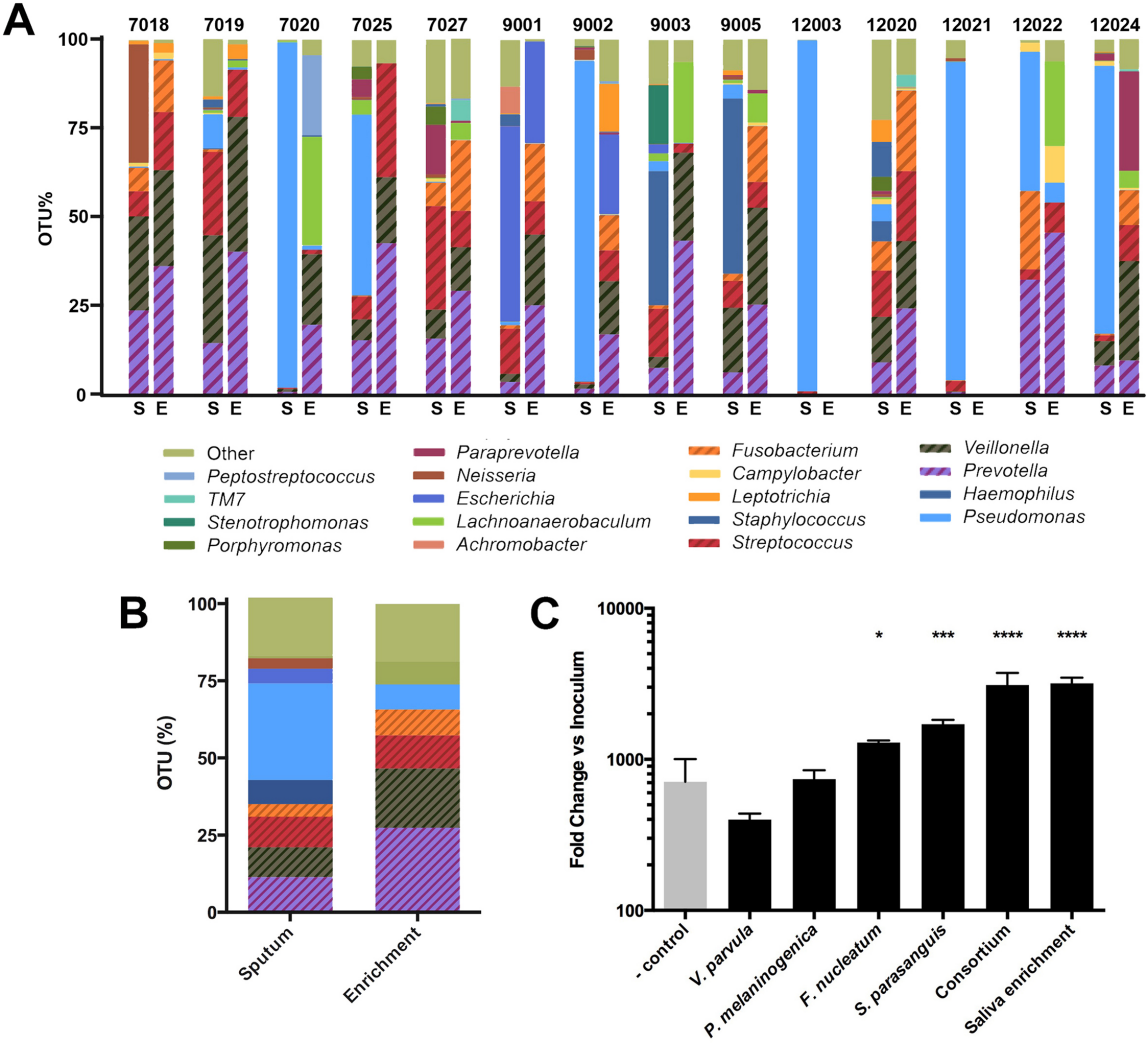
Mucin enrichment of sputum-derived bacterial communities. (A) Taxonomic analysis of bacterial communties in sputum (S) and mucin enrichment cultures (E). A total of 12 enrichment cultures were obtained from 14 cultures seeded with sputum; samples from patient #12003 and #12021 did not grow in enrichment conditions. Faculatative and obligate anaerobes (cross-hatching) capable of mucin fermentation are significantly enriched (p<0.05) during growth on mucins under anoxic conditions. Data shown are genus-level abundances. (B) Mean genus-level taxa abundances across sputum and enrichment samples. (C) Cross-feeding of *P*. *aeruginosa* by sputum-associated mucin-degrading anaerobes. Data shown are mean values +/- SEM from four biological replicates. Asterisks indicate significance versus negative control.

To determine if there exists a fraction of the CF lung microbiota that has the ability to degrade and ferment mucin glycoproteins, we then performed mucin enrichment experiments on expectorated sputum from 14 stable, non-exacerbating CF patients. To do so, a small fraction of sputum was used to inoculate an anaerobic culture medium supplemented with PGM as the sole carbon and nitrogen source. Following anaerobic growth, genomic DNA was isolated from the initial sputum sample and the corresponding enrichment culture followed by bacterial 16S rRNA gene sequencing to identify enriched taxa. If taxa from sputum become enriched in a medium with mucin as the sole carbon source, it would demonstrate the presence of mucin-degradation capacity in the lower airway environment. As expected, sputum microbiota (prior to enrichment) was highly variable between patients (Fig 3A), and *Pseudomonas* made up the highest percentage of sequence reads with a per-patient average of 31.3% across the cohort (Fig 3B). Notably, taxa previously characterized for their mucin-degrading activity (*Prevotella*, *Veillonella*, *Streptococcus* and *Fusobacterium)* also made up 35.1% of the population (11.4%, 9.7%, 9.9% and 4.1% of normalized sequence reads, respectively).

Post-enrichment, sputum-derived communities were predominated by fermentative organisms commonly associated with both the oral cavity and lower airways (Fig 3A and 3B). On average, enrichment communities were composed of 66% of taxa known to have salivary mucin degradation ability (27.4% *Prevotella*, 19.2% *Veillonella*, 10.7% *Streptococcus*, 8.4% *Fusobacterium*) with all other genera present at 4% or below (Fig 3B). *Lachnoanaerobaculum* and *Prevotella* were significantly enriched while *Neisseria*, *Staphylococcus*, and *Pseudomonas* were selected against (*p*<0.05). The relative abundance of mucin-fermenting taxa in the initial sputum samples and their ability to grow on mucin *in vitro* suggests a suitable niche space exists in the CF lung for mucin-fermenting anaerobes.

We then tested representative isolates of the four most abundant mucin-enriched species for their ability to cross-feed *P*. *aeruginosa* PA14 (Fig 3C). Using the co-culture model, mucin degradation by *P*. *melaninogenica* and *V*. *parvula* alone did not stimulate an increase in *P*. *aeruginosa* growth relative to anaerobe-free controls. By contrast, *F*. *nucleatum* and *S*. *parasanguis* both supported a significant increase in PA14 growth; however, final PA14 cell density achieved in the presence of any individual mucin-fermenter was significantly less (*p*<0.0001) than when all four mucin-degraders (*P*.*m*., *V*.*p*., *F*.*n*. and *S*.*p*.*)* were added at an equal density. Notably, the PA14 cell density achieved with this defined, four-species anaerobic consortium was comparable to growth achieved using the undefined enrichment culture (S2 Fig) used in Fig 2. These data demonstrate that while *P*. *aeruginosa* growth can be stimulated by select sputum-enriched anaerobes individually, the collective activity of a mucin-degrading consortium associated with the lower airways results in a significantly higher growth yield.

### CF sputum-derived fermenting organisms produce short-chain fatty acids and amino acids when grown on mucin

**Fig 4:**
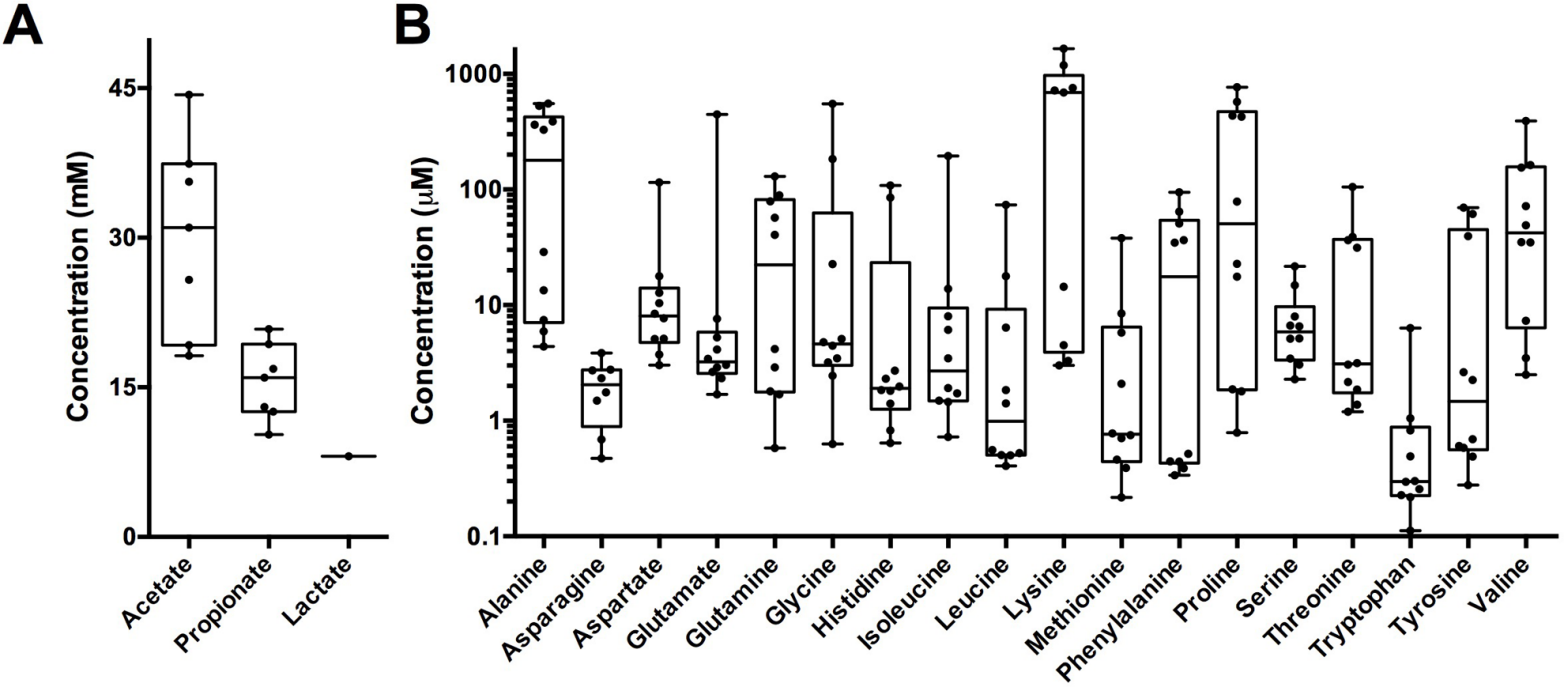
Mucin-enrichment metabolite profiling. (A) short chain fatty acids (n = 8) and (B) amino acids (n = 10) were quantified in mucin enrichment cultures. Arginine and cysteine were unable to be quantified due to their co-elution with other metabolites.

To study the cross-feeding ability of mucin fermenting bacteria in further detail, we then characterized the metabolites that were generated during sputum enrichment. Fermentative anaerobes are known to produce mixed acid metabolites so we quantified a number of organic acids via high performance liquid chromatography (acetate, butyrate, citrate, formate, isobutyrate, isovalerate, ketobutyrate, ketoisovalerate, lactate, 2-methylbutyrate, propionate, succinate and valerate), and quantified amino acid production using gas chromatography. Enrichment cultures generated detectable levels of only three short-chain fatty acids (SCFAs) measured: high amounts of acetate (30.2 +/- 9.7 mM) and propionate (15.4 +/- 3.8 mM) were found in each sample, while only one enrichment sample containing an abundance of *Streptococcus sp*. (#7025, see Fig 3A) produced a detectable concentration of lactate (7.8 mM) (Fig 4A). No other SCFAs assayed were detectable in the mucin-enrichment supernatants.

Not surprisingly, free amino acids were also present in each sample (1.37 +/- 1.32 mM total) (Fig 4B), most likely due to their liberation from the mucin polypeptide backbone. The abundance of amino acids correlates well with previous studies of sputum composition where they were found to be present in appreciable quantities [15]. Altogether, these data demonstrate that acetate, propionate, and amino acids are the major byproducts of mucin fermentation by sputum-derived anaerobes *in vitro*, suggesting that they may be carbon sources that are bioavailable to pathogens *in vivo*.

### Propionate and acetate utilization mutants are defective in mucin cross-feeding co-cultures

**Fig 5:**
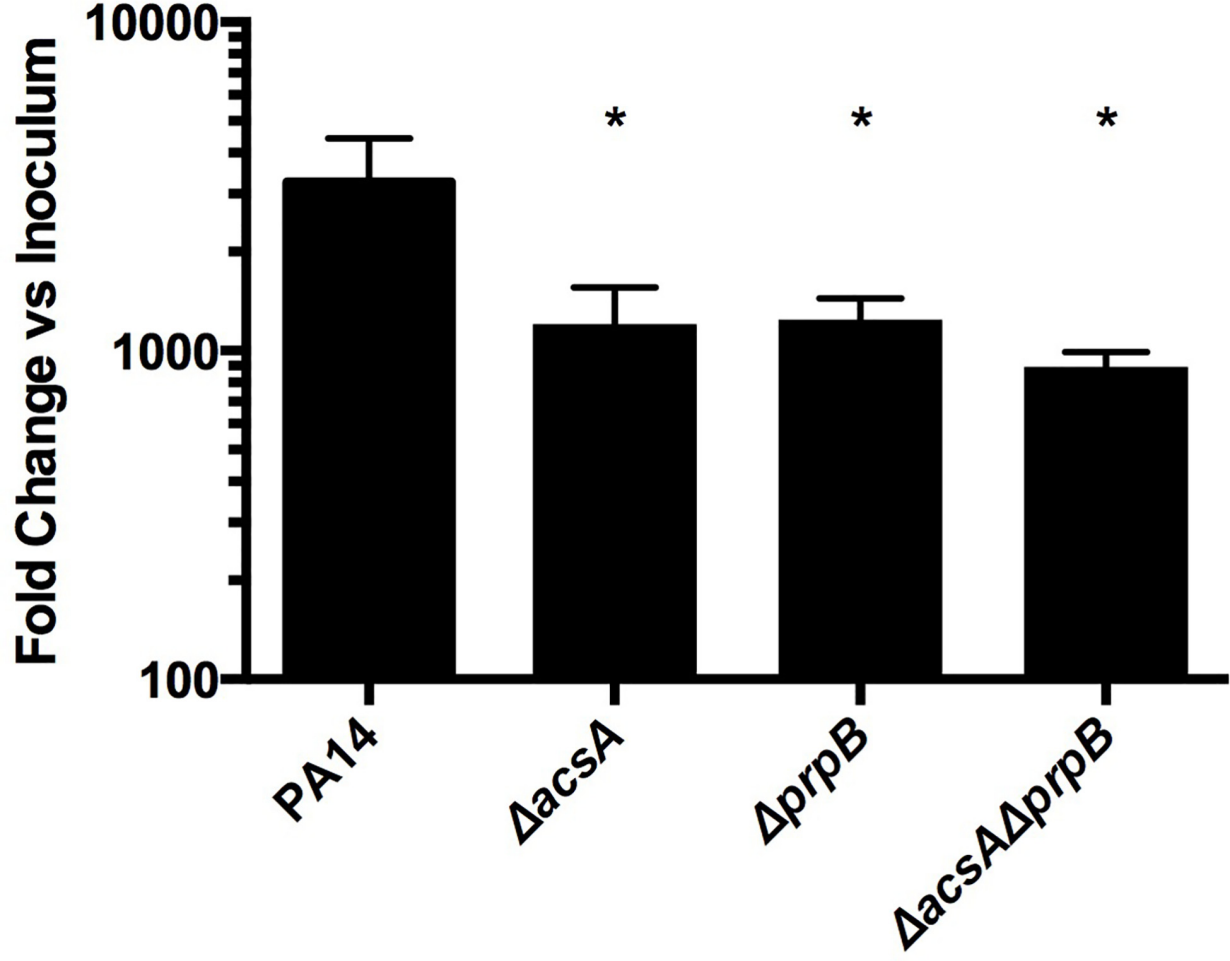
*acsA* and *prpB* mutants are defective in mucin cross-feeding. PA14 mutants lacking genes required for acetate and propionate catabolism show a significant defect in co-culture with mucin-fermenting anaerobes. Asterisks indicate significance relative to PA14.

To assess the contributions of propionate and acetate to *P*. *aeruginosa* growth in our *in vitro* cross-feeding model, we generated mutants lacking the genes encoding AcsA (acetyl-coA synthetase) and PrpB (methylisocitrate lyase) that are required for the catabolism of acetate and propionate, respectively. We also generated a double mutant (Δ*prpB*Δ*acsA*) that was defective in both pathways. Each mutant grew normally on glucose, yet demonstrated a predictable growth limitation when its cognate substrate was provided as the sole carbon source (S3 Fig). Mutants were then tested for their ability to grow in the agar co-culture model with the oral-derived anaerobic consortium (S2 Fig) provided as the mucin-degrading inoculum. Δ*acsA*, Δ*prpB*, and Δ*acsAprpB* demonstrated significant growth defects relative to PA14 (*p<*0.05) when grown in co-culture with mucin fermenters (Fig 5). These data demonstrate that *P*. *aeruginosa* growth is partially dependent on acetate and propionate catabolism in a model bacterial community characteristic of the CF lower airways.

### CF sputum contains short-chain fatty acids and *P*. *aeruginosa* responds to propionate *in vivo*

**Fig 6:**
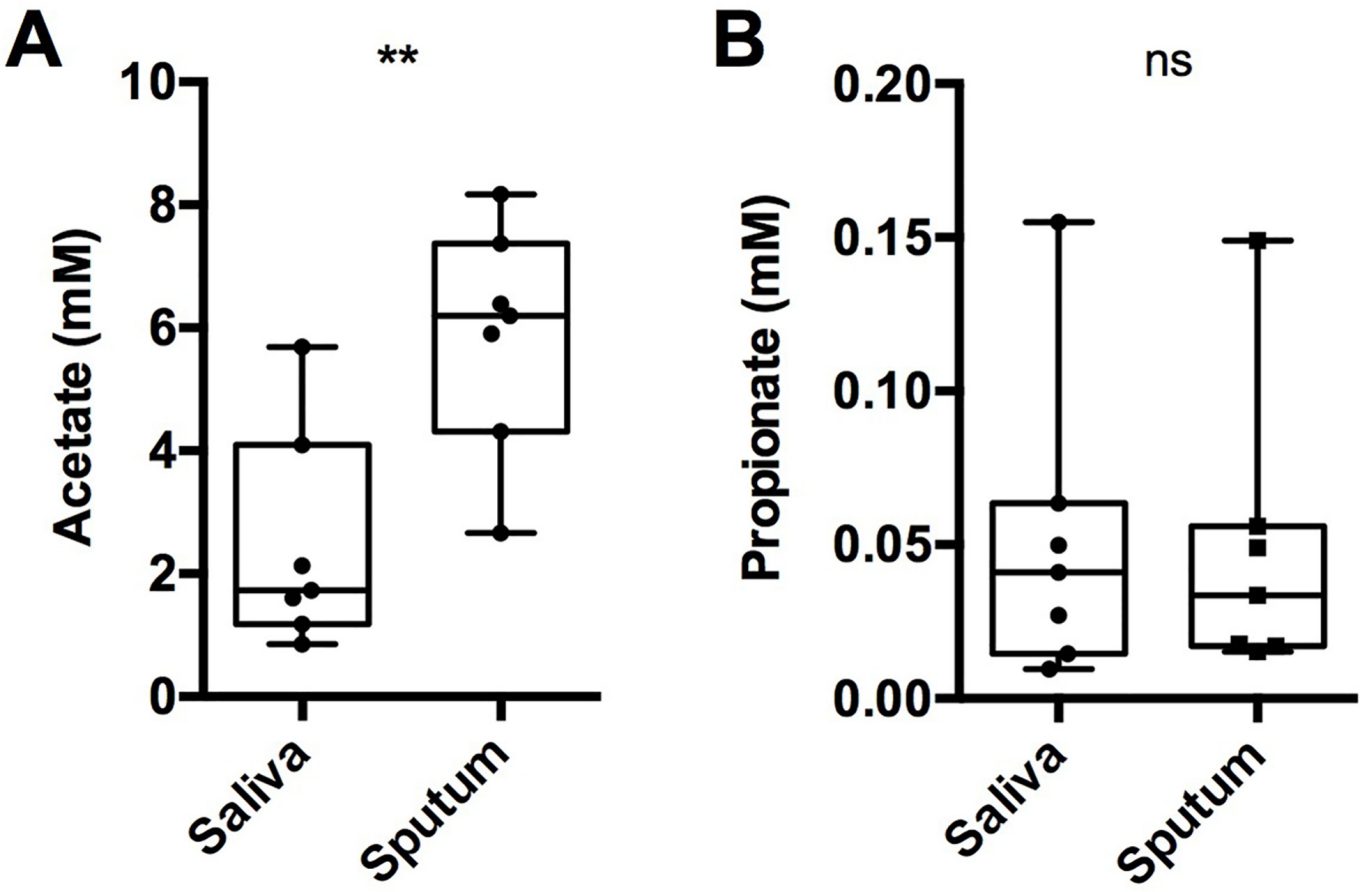
Short chain fatty acids are abundant in the CF airways. (A) Direct measurements of (A) acetate and (B) propionate in paired sputum and saliva samples from CF patients. Acetate concentrations were significantly higher in sputum (**, *p =* 0.004).

Finally, to provide evidence of fermentative activity *in vivo*, we used two complementary techniques to study bacterial metabolism within expectorated sputum: mass spectrometry and quantitative reverse transcription PCR (qRTPCR). First, using gas chromatography mass spectrometry (GC-MS), acetate and propionate were quantified in paired saliva/sputum samples collected from 7 stable CF patients. Given the presence of fermenting taxa and SCFA previously identified in saliva [38], an oral rinse was first performed prior to sample collection to reduce the residual metabolite background from the mouth (see Methods). SCFAs were found at millimolar concentrations (5.9 +/- 1.8 mM and 48.2 +/- 47.2 μM, for acetate and propionate, respectively) in all sputum samples. Despite our washing effort, the saliva sample also contained detectable levels of both acetate and propionate. Acetate concentrations were significantly higher in sputum relative to saliva (5.9 versus 2.4 mM, *p =* 0.004)(Fig 6A). Propionate, on the other hand, showed no significant difference (*p =* 0.89) between sample pairs (Fig 6B). Thus, we cannot rule out propionate contamination from the oral cavity. However, given that the propionate concentration in saliva was not diluted compared to sputum, it is reasonable to approximate that sputum contains a comparable concentration of propionate to the saliva samples. Our results are supported by recent reports of acetate and propionate in CF bronchoalveolar lavage fluid [14,29]. The ratio of propionate to acetate (1:100) in our samples was unexpected given the much higher ratio (1:2) generated during *in vitro* mucin fermentation. This disparity may suggest that propionate is either produced at low levels *in vivo*, or that propionate, over acetate, is preferentially consumed by CF microbiota in a cross-feeding relationship.

**Fig 7:**
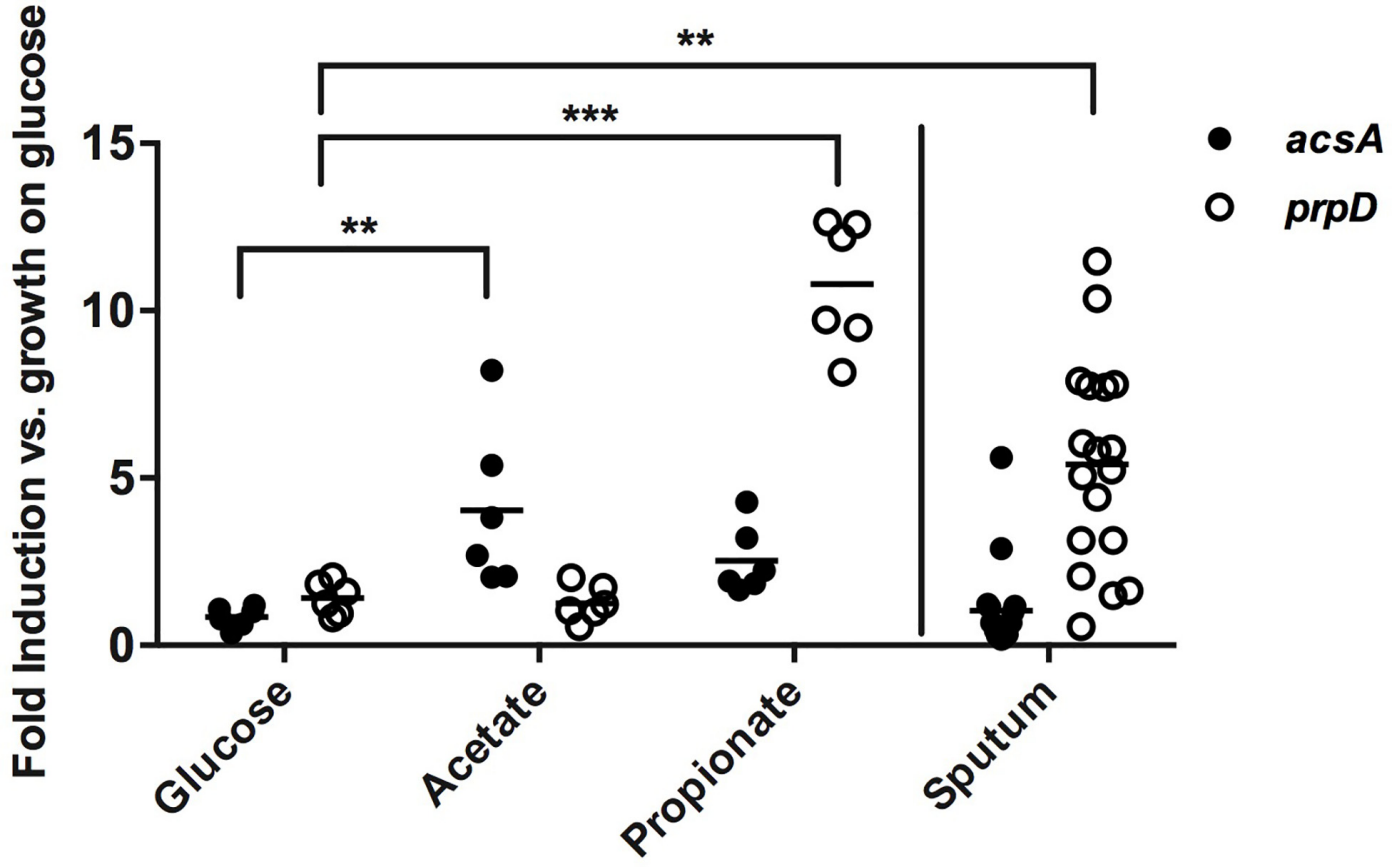
qRT-PCR analysis of *in vivo P*. *aeruginosa* gene expression. *In vitro* expression (left) of *acsA* and *prpD* is upregulated in response to acetate and propionate, respectively. Data shown are biological triplicate measurements for PA14 and JMF5 normalized to *clpX* expression. *In vivo* (right), *prpD* is upregulated (5.4 fold ± 3.1) while *acsA* is not (1.0 fold ± 1.3) (*n = 17*).

As a complement to our GC-MS measurements, we then used qRTPCR to assess whether *Pseudomonas* senses and responds to acetate and/or propionate within the airways. As a proxy of the use of these mucin-derived metabolites by *P*. *aeruginosa*, we targeted the expression of both *acsA* and *prpD* in sputum relative to their expression levels under controlled laboratory conditions. *In vitro*, *acsA* and *prpD* were differentially expressed by both PA14 and JMF5 in the presence of acetate (4.0-fold higher, *p =* 0.01) and propionate (10.8-fold, *p*<0.001), respectively, relative to growth on glucose alone (Fig 7). When compared to *in vitro* cultures, analysis of sputum revealed that *prpD* (5.4-fold, *p =* 0.005) but not *acsA* (no change, *p =* 0.73) was highly expressed. Expression of *prpD* was highly variable, however this was not unexpected given the variable nature of patient samples. These qRTPCR data are consistent with the active catabolism of propionate by *Pseudomonas in vivo*, and may account for the disparity seen in propionate:acetate ratios between sputum and mucin enrichment cultures. If SCFAs were simultaneously produced and consumed by the airway bacterial community, we would expect the concentration of these metabolites to remain low, and genes required for their catabolism to be highly expressed. Though we cannot rule out the possibility of low rates of propionate production *in vivo*, the expression of *prpD* by *Pseudomonas* within sputum is a reliable biomarker of the presence of mixed-acid fermentation byproducts. It is noteworthy that acetate catabolism via AcsA is important to PA14 yields during cross-feeding *in vitro*, yet the qRTPCR data suggest that *acsA* is minimally expressed *in vivo*. This discrepancy may be due to strain-specific differences in PA14 compared to clinical isolates, or may be influenced by unknown environmental cues *in vivo*.

Collectively, data presented here demonstrate the presence of fermentation metabolites and evidence consistent with their catabolism in the airways of CF patients. These results, coupled with previous reports of SCFA in bronchoalveolar lavage fluid [14,29] and degraded mucins in CF sputum [18,39], provide compelling evidence that anaerobic organisms can contribute to CF lung disease by degrading mucins and consequently providing utilizable substrates for opportunistic airway pathogens.

## Discussion

In this study, we investigated the nutritional role of mucins in the growth of *P*. *aeruginosa* in the CF airways. While *Pseudomonas* was found to inefficiently utilize mucins as a carbon source on their own, we determined that mucin fermentation by oral anaerobes can stimulate the growth (10X) of *P*. *aeruginosa* and other opportunistic CF pathogens. Moreover, we revealed that *in vitro* mucin fermentation generated high concentrations of SCFAs and amino acids, which were also abundant and bioavailable for *P*. *aeruginosa* within patient sputum. Together, these results suggest that the high levels of utilizable metabolites present in sputum reported previously [14,15,29] may be derived from bacterial mucin degradation. In this context, fermentative anaerobes aspirated from the oral cavity that become established in the lower airways may play a central role in the progression of CF lung disease.

Expectorated sputum and many of its specific constituents–lipids [15,17], amino acids [16], and modified sugars [40], for example–are known to support bacterial growth *in vitro* and have been studied in detail. Yet, how the majority of these compounds are made available within the CF airways has not been defined. Mucins represent an abundant source of both amino acids and sugars, and play a key role in shaping the microbial community structure of the gastrointestinal tract. However, the process of mucin degradation, and its potential contribution to airway disease has not been addressed in detail. Previous studies have shown that mucins can support the carbon demands of *P*. *aeruginosa in vitro* [41,42]; however, these studies included autoclaved preparations of commercial porcine gastric mucin (PGM) that contain low molecular mass compounds that are readily utilized. In fact, when PGM preparations were filtered and dialyzed in our study (leaving only large, intact glycoproteins) appreciable growth of *P*. *aeruginosa* isolates was not observed.

The near ubiquitous presence of mucin-fermenting anaerobes in sputum [5, 27, 28, this study], coupled with numerous studies demonstrating that the respiratory mucins MUC5B and MUC5AC are degraded in CF patients compared to healthy controls [18,39] supports the idea that bacterial mucin degradation is commonplace within the CF airways. *Streptococcus* sp., for example, which were consistently abundant in mucin-enriched sputum cultures, have been extensively characterized for their ability to degrade salivary mucins [21]. By doing so, oral streptococci modify the nutritional landscape of the oral cavity and stimulate the growth of secondary colonizers [24]. Here we demonstrate that by degrading mucins, commensal anaerobes can also stimulate the growth of opportunistic pathogens found within the respiratory tract, supporting an ecological role for mucin-fermenting anaerobes in the development of CF airway infections.

*In vitro*, degradation of glycan sugars and the polypeptide backbone of mucin by sputum-derived anaerobes generated an abundance of SCFAs and amino acids. Consistent with recent studies [14, 29], SCFAs were also found in CF patient sputum. Because SCFAs serve as a reliable biomarker of fermentative metabolism [43], the universal presence of SCFAs across our patient cohort provides strong supporting evidence for the existence of fermentation within the CF airways. Specifically, our data demonstrate that the fermentation of mucins generates the same metabolites that have been shown to support growth of *Pseudomonas* in sputum [15]. Moreover, the expression of *prpD* in sputum suggests that *P*. *aeruginosa* is both sensing and utilizing propionate *in vivo;* however, the significance of propionate catabolism in disease is not known. While propionate can support growth of *P*. *aeruginosa*, it is also a potent microbial inhibitor [44]. As such, we do not yet know if the *in vivo* degradation of propionate helps to satisfy the carbon requirements of *P*. *aeruginosa* or whether it is being selectively degraded for detoxification purposes.

**Fig 8:**
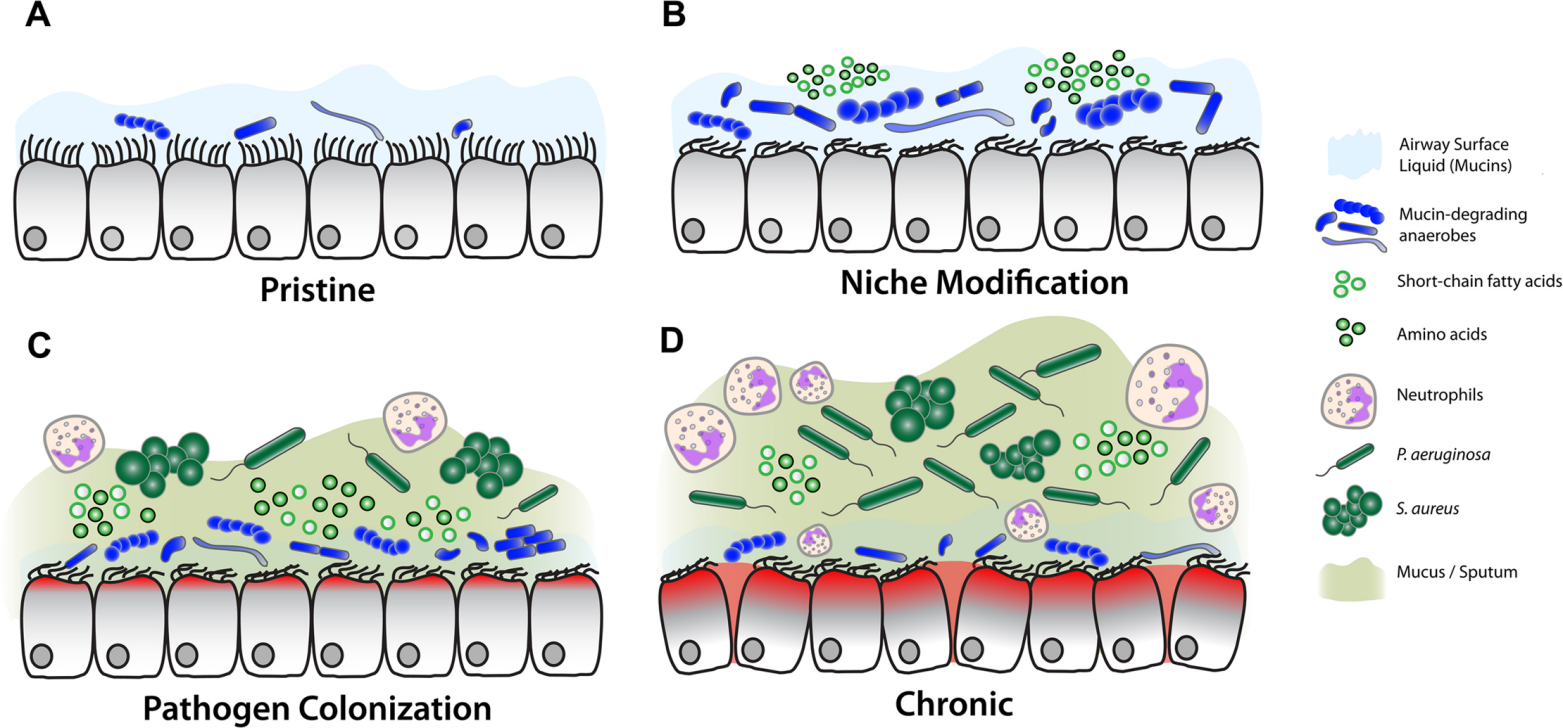
Model for the role of mucin fermenting bacteria in the progression of CF lung disease. (A) In healthy airways or CF newborns, airway surface liquid harbors a low number of aspirated oral bacteria. (B) In CF patients, impaired mucociliary clearance and defective immune responses increase the residence time of oral-associated anaerobes. In turn, their ability to degrade and ferment respiratory mucins modifies the airway environment for secondary colonizers. (C) The abundance of fermentation byproducts within sputum facilitates pathogen colonization, heightened inflammation, neutrophil recruitment and sputum accumulation. (D) In late stages of disease, host inflammatory responses and epithelial damage shape the bioavailable nutrient pool, increasing the abundance of pathogens while oral anaerobes are eliminated by the host and via broad spectrum antibiotic therapies.

Altogether, our data point to a compelling model for the role of oral anaerobes in the development of CF lung disease (Fig 8). In this model, opportunistic pathogens that cannot degrade mucins (*e*.*g*. *P*. *aeruginosa*, *S*. *aureus*) do not become established in the lower airways until mucin-fermenting bacteria have colonized. In healthy subjects, anaerobes that are routinely aspirated into the lower airways encounter functional host defenses and are effectively cleared. In CF, however, impaired mucociliary clearance and defective immunological responses [2] increase the likelihood of oral anaerobe colonization (Fig 8A and 8B). Their increased residence time allows anaerobes to degrade respiratory mucins and condition the lung environment into a niche that is suitable for pathogens to thrive (Fig 8C). Several lines of clinical evidence exist in support of our proposed model: (i) pediatric patients often have asymptomatic primary colonization by oral anaerobes [45,46] prior to the establishment of chronic *P*. *aeruginosa* infections, (ii) routine administration of broad-spectrum antibiotics in the absence of respiratory infection symptoms is an effective therapy to delay the onset of colonization by *P*. *aeruginosa* and reduce the frequency of acute exacerbations [47,48], and (iii) the *in vitro* antibiotic susceptibility of lung pathogens does not always correlate with clinical outcomes [49]. In the latter instance, we propose that antibiotics do not solely target the pathogen, but rather disrupt the complex metabolic interactions that supply them with substrates for growth and stimulate their pathogenicity.

While this work suggests a role for mucin fermenters in the progression to pathogen colonization, it also raises a number of questions. Most importantly, it does not address the role of oral anaerobes or bacterial nutrient acquisition in late stages of CF disease (Fig 8D). In chronic airway infections, bacterial diversity often declines and lung microbiota becomes predominated by *P*. *aeruginosa* [50,51]. Therefore, it is probable that *P*. *aeruginosa* does not rely upon mucin fermentation in late stages, but rather contributions from the host inflammatory response. It is known that chronic airway infections can lead to a ‘leaky’ lung epithelium that allows bulk plasma, with an abundance of bioavailable metabolites, to reach the epithelial surface [52]. Additionally, persistent *P*. *aeruginosa* infections incite a neutrophil-dominant inflammatory response that is associated with increased concentrations of nutrients and proteases in the airway milieu. Human neutrophil elastase, for example, is capable of cleaving mucins glycoproteins into free amino acids, and has been implicated in the progression of lung disease [39]. Given these other potential nutritional sources, it is probable that *P*. *aeruginosa* uses multiple carbon sources *in vivo* as infections progress, and we suspect that pathogens may become independent from mucin fermenters in late stages of CF lung disease.

This study underscores the importance of identifying the underlying microbial ecological dynamics that give rise to CF lung disease progression. In particular, our data warrant further studies of targeted therapies towards fermentative organisms and their metabolisms that may contribute to the establishment and progression of chronic airway disease. In a broader context, the presence of oral anaerobes in other, mucus-rich disease environments where both oral anaerobes and *P*. *aeruginosa* are major players–chronic obstructive pulmonary disease, sinusitis, and ventilator-associated pneumonias–suggests that mucin fermentation and metabolic cross-feeding may be a more widespread phenomenon. We are currently investigating this interspecies dynamic in a range of infectious contexts.

## Methods

### Bacterial strains and culture conditions

Bacterial strains and primers are shown in S1 Table. *P*. *aeruginosa* PA14 and *Staphylococcus aureus* MN8 were obtained from D.K. Newman (California Institute of Technology). *S*. *parasanguis* ATCC 15912 was obtained from M.C. Herzberg (University of Minnesota). *F*. *nucleatum subsp*. *nucleatum* ATCC 25586, *P*. *melaninogenica* ATCC 25845, and *V*. *parvula* ATCC 10790 were obtained from Microbiologics (St. Cloud, MN). Clinical strains of *P*. *aeruginosa*, *B*. *cenocepecia*, *S*. *maltophilia*, and *A*. *xylosoxidans* [53] were isolated from patients enrolled in this study. Aerobes were routinely cultured in Luria Bertani medium or a minimal mucin medium containing 60mM KH_2_PO_4_ (pH 7.4), 90mM NaCl, 1mM MgSO_4_, 15 g L^-1^ porcine gastric mucin (PGM; Sigma), and a trace minerals mix described elsewhere [54]. During preparation, PGM was first autoclaved, dialyzed using a 13 kDa molecular weight cutoff membrane, clarified by centrifugation, followed by passage through a 0.45 μm Millipore syringe filter to sterilize and isolate soluble intact glycoproteins. MUC5B was used in place of PGM where specified, though was used sparingly due to its purification difficulty [55]. Glucose (13mM), NH_4_Cl (60mM), acetate (20mM) and propionate (20mM) were supplemented where specified. MEM essential and non-essential amino acid mixes (Sigma) were added at a final concentration of 0.5X the manufacturer suggested concentration. *S*. *parasanguis*, *F*. *nucleatum*, *P*. *melaninogenica*, and *V*. *parvula* were cultured in Brain-Heart Infusion media supplemented with hemin (0.25 g L^-1^), vitamin K (0.025 g L^-1^) and laked sheep’s blood (5% vol/vol)(BHI-HKB). Growth was monitored in 96-well plates (Corning) in 250 μL volumes using a BioTek Synergy H1 plate reader. For enrichment growth, approximately 100 μL of sputum was then used to inoculate minimal mucin medium under anoxia (95% N_2_, 5% CO_2_) and the remainder was frozen at -80°C. Following 48h of incubation at 37°C, 100 μL of culture was used to inoculate a second anaerobic culture tube and allowed to grow for 48h at 37°C. Genomic DNA was isolated from both the initial sputum sample and enrichment cultures, and bacterial community composition was determined using 16S rRNA gene sequencing.

### Patient cohort and sample collection

Forty-eight adult participants with CF were recruited at the University of Minnesota Adult CF Center. Inclusion criteria were a positive diagnosis of CF and ability to expectorate sputum. For enrichment cultures, sputum was expectorated following an oral rinse into 50mL conical tubes, placed on ice, and processed within three hours. Sputum used for qRTPCR analysis was placed in RNALater (Sigma) immediately following expectoration, and stored at -80°C. For metabolite analysis (n = 7), subjects first performed an oral rinse, followed by paired saliva and sputum collection and immediate storage at -80°C. To obtain clinical isolates, sputum aliquots were cultured on Pseudomonas Isolation Agar (Oxoid) for 72 hours at 37°C. Colonies were screened using PCR, sequenced to confirm identity, and stored in 15% glycerol at -80°C.

### DNA extraction and 16S community analysis

Sputum and enrichment cultures were thawed to room temperature, and 500 μL of each sample was used for genomic DNA extraction using the PowerSoil DNA isolation kit (MoBio, Carlsbad, CA). Purified DNA was submitted to the University of Minnesota Genomics Center (UMGC) for 16S library preparation using a two-step PCR protocol described previously [56]. A defined mock community was also submitted for sequencing, as were water and reagent controls that did not pass quality control step due to 16S rRNA gene content below detection thresholds. Raw sequence reads were obtained from UMGC and analyzed using a QIIME [57] pipeline developed by the UMGC. The average number of reads per sample after filtering and taxonomic assignment was 2.2 x 10^5^, with the minimum and maximum reads per sample of 5.6 x 10^3^ and 5.1 x 10^5^, respectively. Read pairs were stitched together and 16S amplicon primers were removed using PandaSeq (version 2.7)[58]. Fastq files were merged and sequence IDs converted to QIIME format using a custom perl script. Chimeric sequences were detected using the QIIME (version 1.8.0) script identify_chimeric_seq.py function, using the usearch61 method. Open reference OTU picking was performed using the pick_open_reference_otus.py script, using the usearch61 method and the Greengenes 13_8 16S rRNA reference database [59] clustered at 97% similarity. MetaStats was used to detect differentially abundant features of the mucin-enriched bacterial community [60].

### *In vitro* cross-feeding

Mucin fermentation cultures (diluted 1/100) were used to inoculate 2 mL of mucin (15g L^-1^) minimal medium in molten 1.0% agar at 50°C in a glass culture tube under anoxic conditions (Fig 2A). Mucin fermentation cultures contained one of the following: (1) individual anaerobic species (*V*. *parvula*, *F*. *nucleatum*, *P*. *melaninogenica*, or *S*. *parasanguis*) grown from single colony picks, grown overnight in anaerobic BHI-HKB broth and washed twice with PBS; (2) a defined four-species anaerobic consortium (*V*.*parvula*, *F*. *nucleatum*, *P*. *melaninogenica*, and *S*. *parasanguis* together) prepared as described above; or (3) a saliva-derived mucin-fermenting bacterial community (S2 Fig) revived from glycerol freezer stocks and passaged twice in mucin minimal medium. Upon solidification of the mucin-fermenting fraction, an additional 1 mL of molten minimal medium agar (without mucin) was inoculated with a 1/1000 dilution of an overnight culture of *P*. *aeruginosa* (or other strains where specified). Inoculum sizes are shown in S2 Table. Samples were then poured over the mucin fermenting community fraction and allowed to solidify. Tubes without mucin-fermenters were used as negative controls. After solidification, co-cultures were placed at 37°C for 48 h or other specified time points. Agar plugs were then removed from the upper phase and homogenized by pipetting in 10 mL of phosphate buffered saline. Colony forming units per tube were determined by serial dilution and plating on LB agar.

### Generation of clean-deletion mutants

Unmarked deletions were generated for the genes *prpB* (PA14_53940) and *acsA* (PA14_52800) in the PA14 wild-type background. Flanking regions (~1kb in length) containing the first and last codons of for *acsA* and *prpB* were generated using primers listed in S1 Table. The flanking regions and the deletion vector pSMV8 [61] (linearized by digestion with XhoI and SpeI) were assembled by Gibson assembly [62]. The resulting plasmid was transformed into *E*. *coli* WM3064 [61] and mobilized into PA14 by conjugation. Single recombinants were selected on LB agar containing 50 μg mL^-1^ gentamicin. Double recombinants were selected for on LB agar containing 6% sucrose. Potential *prpB* and *acsA* mutants, or *prpBacsA* double mutants were identified by PCR, and markerless deletions were confirmed by sequencing.

### Metabolite quantification

Targeted quantification of short-chain fatty acids was performed via high performance liquid chromatography (HPLC). The system consisted of a Shimadzu SCL-10A system controller, LC-10AT liquid chromatograph, SIL-10AF autoinjector, SPD-10A UV-Vis detector, and CTO-10A column oven. Separation of compounds was performed with an Aminex HPX-87H guard column and an HPX-87H cation-exchange column (Bio-Rad [Hercules, CA]). The mobile phase consisted of 0.05 N H_2_SO_4_, set at a flow rate of 0.5 mL min^-1^. The column was maintained at 50°C and the injection volume was 50 μL. Amino acid and metabolite (acetate and propionate) quantification from enrichment supernatants were performed by Millis Scientific, Inc. (Baltimore, MD) using liquid chromatography-mass spectrometry and gas chromatography-mass spectrometry (GC-MS). Samples for amino acid quantification were spiked with 1 μL of uniformly labeled amino acids (Cambridge Isotope Labs) and derivatized using AccQ-Tag reagent (Waters Corp.) for 10 min at 50°C. A Waters Micromass Quatro LC-MS interfaced with a Waters Atlantis dC18 (3 μm 2.1x100 mm) column was used. Reverse-phase LC was used for separation (mobile phases A:10mM ammonium formate in 0.5% formic acid, B:methanol) with a constant flow rate (0.2 mL min^-1^) and a column temperature of 40°C. Electrospray ionization was used to generate ions in the positive mode and multiple reaction monitoring was used to quantify amino acids. Samples (~100 μL) for acetate and propionate quantification were first diluted (150 μL water), spiked with internal standards (10 μL of 1000ppm acetate [^13^C_2_] and 1000 ppm propionate [^13^C_1_]) and acidified using 2 μL of 12N HCl. After equilibration at 60°C for 2h, carboxen/ polydimethylsiloxane solid phase microextraction (SPME) fiber was used to adsorb the headspace at 60°C for 30min. Acids were then desorbed into the gas chromatograph inlet for 2 min. A 30 m x 0.32 mm ID DB-624 column attached to a Thermo Electron Trace gas chromatograph with helium carrier gas (2.0 mL min^-1^) was used for separation of analytes. A Waters Micromass Quatro GC mass spectrometer was used for detection and quantification of target ions. Significance between sputum and saliva samples were determined by paired Student’s t-test.

### RNA isolation, purification, and qRTPCR

To quantify *P*. *aeruginosa* gene expression *in vivo*, sputum (n = 17) was expectorated into RNAlater and immediately frozen to preserve the gene expression profile. Frozen sputum was thawed in TriZol (Life Technologies), homogenized using ceramic beads and purified according to the manufacturer’s protocol. RNA was concentrated using the Clean & Concentrator kit (Zymo) and de-salted using Turbo DNA-free (Life Technologies). Bacterial RNA was enriched (only in sputum samples) using the MicrobEnrich kit (Life Technologies), and purity was confirmed using Qubit (LifeTechnologies) spectrophotometry. qRTPCR was performed as previously described [63]. Briefly, DNA was reverse transcribed from 1 μg of total RNA using the iScript cDNA synthesis kit (BioRad). cDNA was then used a template for quantitative PCR on an iQ5 thermocycler (BioRad) using iTaq Universal SYBR Green Super Mix (BioRad). Triplicate measurements were made on each sputum sample. For control cultures, *P*. *aeruginosa* was grown in 4-morpholinepropanesulfonic acid (MOPS) minimal medium to an OD600 of~0.6 supplemented with glucose (12mM), acetate (20mM), propionate (20mM), or acetate + propionate (20mM each) where specified. After growth, cells were harvested by centrifugation, frozen at -80°C, and RNA was extracted as described above. Primer pairs are listed in S1 Table. For all primer sets, the following cycling parameters were used: 94°C for 3 min followed by 40 cycles of 94°C for 60s, 55°C for 45s, and 72°C for 60s. Primer efficiencies were tested for *clpX* (91.6%), *acsA* (91.6%) and *prpD* (96.8%). Relative RNA values were calculated from the Ct values reported and the experimental primer efficiencies, and were normalized to the expression of *clpX*. *clpX* was compared to *oprI* values to ensure constitutive expression levels. Significance between treatments was determined by two-tailed unpaired Student’s t-test.

### Nucleotide sequence accession numbers

16S rRNA gene sequences generated as part of this study were deposited as fastq files in NCBI GenBank under BioProject accession number SRP067035.

### Ethics statement

These studies were approved by the Institutional Review Board at the University of Minnesota (UMN IRB nos. 1401M47262 and 1404M49426). All subjects (adults) provided informed written consent prior to sample collection.

## Supporting Information

S1 Fig *P*. *aeruginosa* growth on MUC5B and porcine gastric mucins (PGM). PA14 was inoculated into a culture containing 3 g L^-1^ PGM or purified MUC5B and allowed to grow at 37°C and shaken continuously. Colony forming units were determined at both 24h and 48h and densities were found to be comparable, though slightly less dense when growing with MUC5B as the sole carbon and nitrogen source, confirming the inability to utilize mucin glycoproteins as a growth substrate.
(PDF)

S2 Fig Taxonomic composition of the mucin-enriched, saliva derived bacterial community used in this study. Data shown are genus-level relative abundances.
(PDF)

S3 Fig **(A)** Mutations in propionate catabolism (Δ*prpB*), acetate catabolism (Δ*acsA*) or both (Δ*prpB*Δ*acsA*), result in growth defects when cultured in the presence of their cognate substrate(s) as the sole carbon source. **(B)** Mutations in propionate and acetate catabolism pathways showed no general growth defects when grown in LB.
(PDF)

S1 Table Oligonucleotide primers used in this study. (PDF)

S2 Table Inoculum sizes for mucin cross-feeding co-cultures. (PDF)
