# Supplementary material for: Evidence and Role for Bacterial Mucin Degradation in Cystic Fibrosis Airway Disease
Source: PLoS Pathog. 2016 Aug 22;12(8):e1005846. doi: 10.1371/journal.ppat.1005846 (PMC4993466; doi:10.1371/journal.ppat.1005846)
Supplement: S1 Fig — PA14 was inoculated into a culture containing 3 g L-1 PGM or purified MUC5B and allowed to grow at 37°C and shaken continuously. Colony forming units were determined at both 24h and 48h and densities were found to be comparable, though slightly less dense when growing with MUC5B as the sole carbon and nitrogen source, confirming the inability to utilize mucin glycoproteins as a growth substrate. (PDF) [file ppat.1005846.s001.pdf]

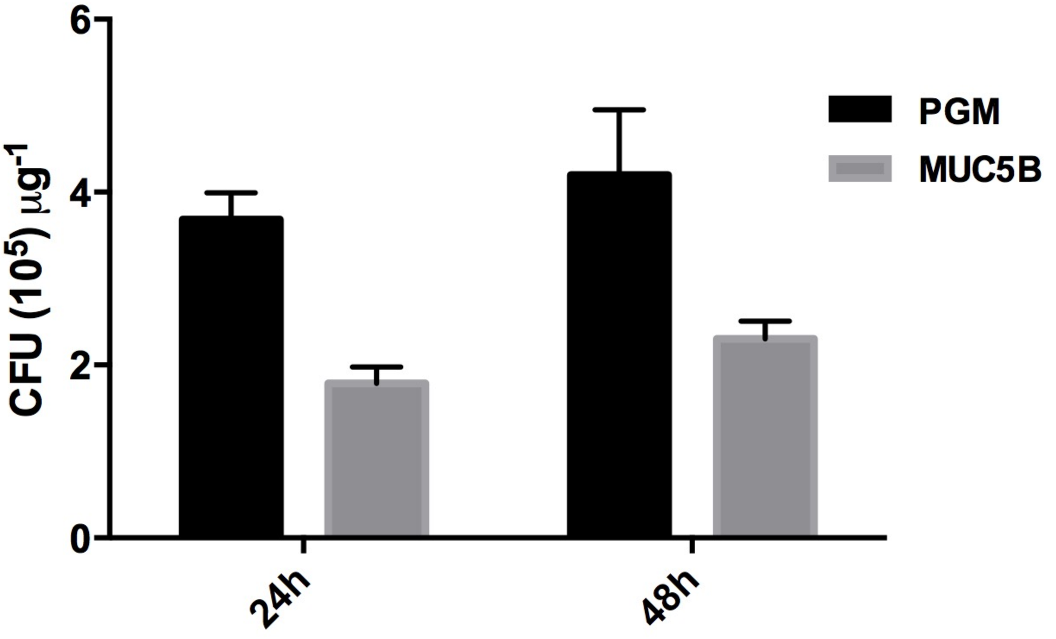

Figure S1. *P. aeruginosa* growth on MUC5B and porcine gastric mucins (PGM). PA14 was inoculated into a culture containing 3 g L^{-1} PGM or purified MUC5B and allowed to grow at 37°C and shaken continuously. Colony forming units were determined at both 24h and 48h and densities were found to be comparable, though slightly less dense when growing with MUC5B as the sole carbon and nitrogen source, confirming the inability to utilize mucin glycoproteins as a growth substrate. Data are expressed as mean +/- SEM (n=3) of CFU μg^{-1} of mucin as determined by Qubit spectrophotometry using bovine serum albumin as a standard.
