# Supplementary material for: Evidence and Role for Bacterial Mucin Degradation in Cystic Fibrosis Airway Disease
Source: PLoS Pathog. 2016 Aug 22;12(8):e1005846. doi: 10.1371/journal.ppat.1005846 (PMC4993466; doi:10.1371/journal.ppat.1005846)
Supplement: S2 Fig — Data shown are genus-level relative abundances. (PDF) [file ppat.1005846.s002.pdf]

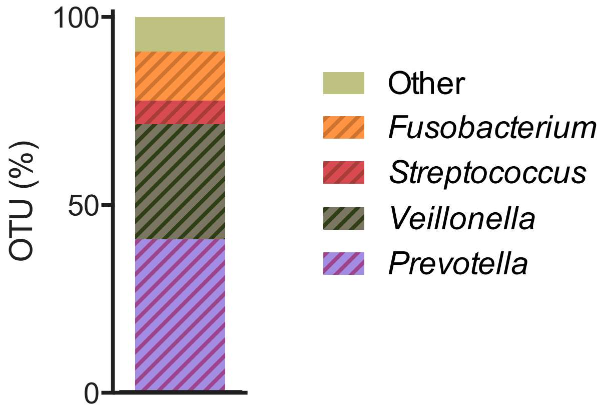

Figure S2. Taxonomic composition of the mucin-enriched, saliva derived bacterial community used in this study. Data shown are genus-level abundances.
