# Supplementary material for: Evidence and Role for Bacterial Mucin Degradation in Cystic Fibrosis Airway Disease
Source: PLoS Pathog. 2016 Aug 22;12(8):e1005846. doi: 10.1371/journal.ppat.1005846 (PMC4993466; doi:10.1371/journal.ppat.1005846)
Supplement: S3 Fig — (A) Mutations in propionate catabolism (ΔprpB), acetate catabolism (ΔacsA) or both (ΔprpBΔacsA), result in growth defects when cultured in the presence of their cognate substrate(s) as the sole carbon source. (B) Mutations in propionate and acetate catabolism pathways showed no general growth defects when grown in LB. (PDF) [file ppat.1005846.s003.pdf]

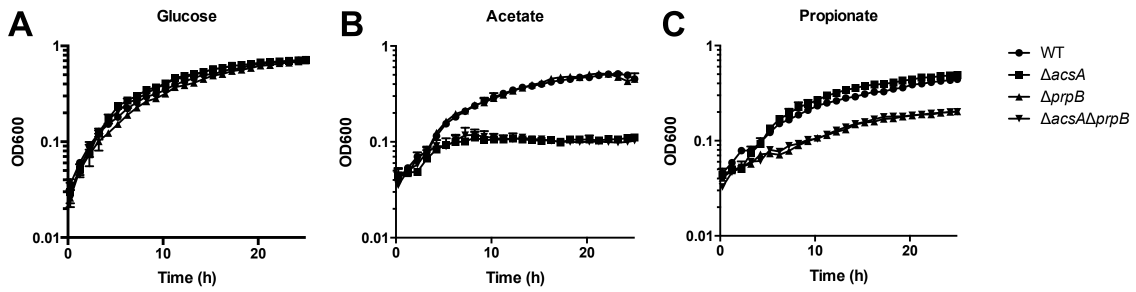

Fig S3. PA14 and markerless deletions in *acsA* and *prpD* grow normally in glucose but exhibit growth defects on acetate and propionate, respectively, when provided as a sole carbon source.
