# Supplementary material for: Evidence and Role for Bacterial Mucin Degradation in Cystic Fibrosis Airway Disease
Source: PLoS Pathog. 2016 Aug 22;12(8):e1005846. doi: 10.1371/journal.ppat.1005846 (PMC4993466; doi:10.1371/journal.ppat.1005846)
Supplement: S1 Table — (PDF) [file ppat.1005846.s004.pdf]

Table S1. Strains and oligonucleotides used in this study.

Strain	Source
Pseudomonas aeruginosa PA14	DK Newman
P. aeruginosa PA14 Δ*acsA*	This study
P. aeruginosa PA14 Δ*prpB*	This study
P. aeruginosa PA14 Δ*acsA* Δ*prpB*	This study
P. aeruginosa JMF1 (clinical isolate)	This study
P. aeruginosa JMF2 (clinical isolate)	This study
P. aeruginosa JMF3 (clinical isolate)	This study
P. aeruginosa JMF4 (clinical isolate)	This study
P. aeruginosa JMF5 (clinical isolate)	This study
P. aeruginosa JMF6 (clinical isolate)	This study
E. coli WM3064	[61]
Veillonella parvula	ATCC 10790
Fusobacterium nucleatum subsp. *nucleatum*	ATCC 25586
Prevotella melaninogenica	ATCC 25845
Streptococcus parasanguis	ATCC 15912
Burkholderia cenocepecia RCH181	This study
Stenotrophomonas maltophilia RCH110	This study
Achromobacter xylosoxidans MN001	[53]
Staphylococcus aureus MN8	DK Newman

qRTPCR oligonucleotide primers

Target Gene	Primer	Sequence (5'-3')	Primer Efficiency %
clpX	*clpX* F	CCTGTGCAATGACATCATCC	91.6
	clpX R	TTCTTGTCACGCTGGTTGAG	
oprI	*oprI* F	TGCGATCACCACCTTCTACT	
	oprI R	GCCCAGAGCCATGTTGTAC	
prpD	*prpD* F	GCAGTGGGGTTTCTACGATG	96.8
	prpD R	CTTGAACAGCACGTTTTCCA	
acsA	*acsA* F	TGTTCGAGGGCGTACCGAACT	91.6
	acsA R	AGCAGACGCAGGCTGGAA	

16S gene sequencing indexing primers

Amplification Step	Primer	Sequence (5'-3')
Primary	V3F Nextera	TCGTCGGCAGCGTCAGATGTGTATAAGAGACAGCTACGGGAGGCAGCAG
	V5R Nextera	GTCTCGTGGGCTCGGAGATGTGTATAAGAGACAGCCGTCAATTCMTTTRAGT
Indexing	Index F	AATGATACGGCGACCACCGAGATCTACACXXXXXXXXTCGTCGGCAGCGTC [a]
	Index R	GATXXXXXXXXGTCTCGTGGGCTCGG [a]

Deletion primers

Gene	Primer	Sequence (5'-3')
acsA	UpFwd	ATTGGGTACCGGGCCCCCCCTCGAGTCATCCCGCCATCGATCGTG
	UpRev	CGCAGACATGGCTTTAACCTC
	DnFwd	GAGGTTAAAGCCATGTCTGCGTGACTTCCGCCTGATCCGA
	DnRev	CGGTGGCGGCCGCTCTAGAACTAGTCATCGGTCTTGAGGAAGCCATCT
prpB	UpFwd	ATTGGGTACCGGGCCCCCCCTCGAGTTTCCACCGGGATGCCGATTT
	UpRev	CTCGATGCTCAGGCGTTCTTCTTGGTAAGGGACGTCTGGCTCAT
	DnFwd	ATGAGCCAGACGTCCCTTACCAAGAAGAACGCCTGAGCATCGAG
	DnRev	CGGTGGCGGCCGCTCTAGAACTAGTTATCGGCGTGAACAGCTTGGT

[a] X marks the position of 8bp indices
