# Supplementary material for: Evidence and Role for Bacterial Mucin Degradation in Cystic Fibrosis Airway Disease
Source: PLoS Pathog. 2016 Aug 22;12(8):e1005846. doi: 10.1371/journal.ppat.1005846 (PMC4993466; doi:10.1371/journal.ppat.1005846)
Supplement: S2 Table — (PDF) [file ppat.1005846.s005.pdf]

Table S2. Starting inoculum density for agar co-culture experiments. Starting cultures were prepared by diluting overnight LB cultures 1/1000.

Strain	CFU / tube
P. aeruginosa PA14	**4.6×10^{5}**
P. aeruginosa JMF1	**1.0×10^{6}**
P. aeruginosa JMF2	**7.8×10^{5}**
P. aeruginosa JMF3	**1.9×10^{5}**
P. aeruginosa JMF4	**1.8×10^{5}**
P. aeruginosa JMF5	**5.1×10^{5}**
P. aeruginosa JMF6	**3.3×10^{5}**
B. cenocepecia RCH181	**4.4×10^{4}**
A. xylosoxidans MN001	**1.5×10^{6}**
S. aureus MN8	**4.2×10^{5}**
S. maltophilia RCH110	**1.5×10^{6}**
